# Body Mass Index and Late Adverse Outcomes after a Carotid Endarterectomy

**DOI:** 10.3390/ijerph20032692

**Published:** 2023-02-02

**Authors:** Danka Vukašinović, Miloš Maksimović, Slobodan Tanasković, Jelena M. Marinković, Đorđe Radak, Jadranka Maksimović, Isidora Vujčić, Nebojša Prijović, Hristina Vlajinac

**Affiliations:** 1 Institute of Hygiene and Medical Ecology, Faculty of Medicine, University of Belgrade, 11000 Belgrade, Serbia; 2 Vascular Surgery Clinic, “Dedinje” Cardiovascular Institute, 11000 Belgrade, Serbia; 3 Faculty of Medicine, University of Belgrade, 11000 Belgrade, Serbia; 4 Institute of Medical Statistics and Informatics, Faculty of Medicine, University of Belgrade, 11000 Belgrade, Serbia; 5 Institute of Epidemiology, Faculty of Medicine, University of Belgrade, 11000 Belgrade, Serbia; 6 Clinic of Urology, University Clinical Center of Serbia, 11000 Belgrade, Serbia

**Keywords:** body mass index, carotid endarterectomy, late outcomes, overweight, obesity

## Abstract

A cohort study was conducted to examine the association of an increased body mass index (BMI) with late adverse outcomes after a carotid endarterectomy (CEA). It comprised 1597 CEAs, performed in 1533 patients at the Vascular Surgery Clinic in Belgrade, from 1 January 2012 to 31 December 2017. The follow-up lasted four years after CEA. Data for late myocardial infarction and stroke were available for 1223 CEAs, data for death for 1305 CEAs, and data for restenosis for 1162 CEAs. Logistic and Cox regressions were used in the analysis. The CEAs in patients who were overweight and obese were separately compared with the CEAs in patients with a normal weight. Out of 1223 CEAs, 413 (33.8%) were performed in patients with a normal weight, 583 (47.7%) in patients who were overweight, and 220 (18.0%) in patients who were obese. According to the logistic regression analysis, the compared groups did not significantly differ in the frequency of myocardial infarction, stroke, and death, as late major adverse outcomes (MAOs), or in the frequency of restenosis. According to the Cox and logistic regression analyses, BMI was neither a predictor for late MAOs, analyzed separately or all together, nor for restenosis. In conclusion, being overweight and being obese were not related to the occurrence of late adverse outcomes after a carotid endarterectomy.

## 1. Introduction

According to the World Health Organization (WHO), in 2016, almost 60% of adults in the WHO European Region were overweight and obese [1]. In Serbia, according to the latest health survey from 2019, the results were similar to those in Europe: 36.3% of adults were overweight, while 20.8% were obese [2]. Being overweight and obese are the fourth most common risk factors for noncommunicable diseases, including cancers, cardiovascular and cerebrovascular diseases, type 2 diabetes mellitus, and chronic respiratory disorders [1,2,3,4,5,6]. They have also been related to perioperative mortality after vascular surgery [7,8]. In addition, some studies have identified obesity as an independent risk factor for carotid plaque destabilization [9], although others showed no relationship between obesity and carotid artery stenosis [10].

Extracranial carotid disease accounts for approximately 18–25% of ischemic strokes [11,12]. Carotid endarterectomy, as a stroke preventive procedure, carries some risk for periprocedural and postprocedural complications that must be considered in the overall assessment of its safety and efficacy.

Data on the association between obesity and CEA adverse outcomes are inconsistent. In some of the investigations [13,14], there was no association between obesity and stroke or death as early adverse outcomes after CEA. Still, there are also studies in which perioperative mortality after the intervention was higher in obese patients [15,16], as well as studies in which mortality and stroke were significantly lower in obese patients [17,18], which corresponds to the so-called “obesity paradox”. Most of these studies referred to early adverse outcomes.

The aim of the present study was to examine the association of increased BMI (overweight and obesity) with late adverse outcomes after CEA.

## 2. Materials and Methods

As previously described in detail [14], this cohort study comprised 1533 patients in whom 1597 CEAs were performed at the University Vascular Surgery Clinic in Belgrade from 1 January 2012 to 31 December 2017. Patients who previously had massive cerebrovascular insult and severe neurological damage, patients with CEA and simultaneous coronary artery bypass grafting, patients with severe cardiac comorbidity, and patients with severe renal insufficiency were not included in the study.

The body mass index (BMI) was calculated as the weight in kg divided by the height in m^2^. For the purpose of the present study, patients were categorized into four categories: underweight (<18.5), normal weight (BMI = 18.5–24.9), overweight (BMI = 25.0–29.9), and obese (BMI ≥ 30) [19]. The BMI status before the operation was used in the analyses of all CEA outcomes.

From all patients, we obtained data on some demographic characteristics, body height and weight, smoking, comorbidities, some laboratory findings at admission, family history of cardiovascular diseases, characteristics of carotid disease, the operative data, and the preoperative therapy, as well as the hospital discharge therapy. All CEAs were performed under general anesthesia.

The patients were monitored for the first 30 postoperative days in order to determine whether there were any differences in early adverse outcomes, according to BMI; the results obtained were previously presented [14].

Follow-up was performed four years after CEA. Patients who did not come to the control examinations at the Clinic where they were operated on were contacted by phone. In the phone interview performed with the study participant or a member of their family, the following data were collected: data of patients’ survival and cause of death, if known, neurological and cardiac events, and their time of occurrence, as well as the data of the patients’ last duplex ultrasound control and possible restenosis. Only the first event of each outcome was included in the analysis. Data for myocardial infarction and stroke were available for 1223 CEAs, data for death for 1305 CEAs, and data for restenosis for 1162 CEAs.

The Ethics Committee of the Faculty of Medicine, Belgrade approved this study (No. 1322/XII-1).

### Statistical Analysis

The categorical variables were presented as numbers and percentages, and continuous variables were presented as the means and standard deviations. The CEAs in patients who were overweight and obese were compared separately with the CEAs in patients with a normal weight. Univariate and multivariate logistic regressions were used in the analysis. All variables that were, according to univariate analysis, associated with overweight or obesity at a level of *p* ≤ 0.10 were included in the model of multivariate analysis. Cox regression analysis was used to assess the hazard ratio of the late adverse outcomes (myocardial infarction, stroke, and death, separately and all together as late major adverse outcomes—MAOs) in patients who were overweight and obese compared to those with a normal weight. We analyzed whether being overweight and having obesity were predictors of restenosis using logistic regression analysis, since we had no data on the time when restenosis occurred. The selection method was backward Wald. All *p* values were based on a two-tailed test, and *p* < 0.05 was considered significant. In order to determine whether there was any correlation between the level of the BMI and the late adverse outcomes, the ROC curve analysis was also used. In addition, we analyzed the correlation between the independent variables included in the study. Follow-up data concerning stroke, myocardial infarction, and death, separately and together, were analyzed by the Kaplan–Meier test. The Statistical Package for Social Sciences (SPSS), version 23, was used for the analysis.

## 3. Results

Out of the 1223 CEAs, for which the data of late MAOs (myocardial infarction, stroke, and death all together) were obtained, 413 (33.8%) were performed in patients with a normal BMI, 583 (47.7%) in patients who were overweight, and 220 (18.0%) in patients with obesity. The number of CEAs in patients who were underweight (seven CEAs—0.5%) was too small to be included in further analysis.

In comparison to the patients with a normal BMI (Table 1), the patients who were overweight were more frequently males (*p* < 0.01), and, in their personal history, they more frequently had an aortocoronary bypass—ACB (*p* ≤ 0.10), aneurysmatic disease (*p* ≤ 0.10), and non-insulin-dependent diabetes mellitus—NIDDM (*p* < 0.05). Compared to the patients with a normal BMI, the patients with obesity were younger (*p* < 0.05), and in their personal history, they more frequently had a myocardial infarction (*p* < 0.01) and NIDDM (*p* < 0.001) and had a more frequent family history of cardiovascular diseases—CVD (*p* ≤ 0.10). Moreover, they used OAC therapy more frequently before the operation (*p* < 0.05) and had higher values of triglycerides at hospital admission (*p* < 0.05).

According to the data presented in Table 2, there were no significant differences between the groups in terms of the characteristics of the carotid disease and in the type of surgery; however, in the patients with obesity, the clump duration was shorter than in those with a normal BMI (*p* ≤ 0.10). Statistically significant differences were found for some drugs prescribed at the time of discharge: ACEI were prescribed more frequently in patients who were overweight compared to those with a normal weight (*p* < 0.01), and OAC were prescribed more frequently in patients with obesity than patients with a normal BMI (*p* < 0.05). Moreover, in comparison to the patients with a normal weight, higher doses of statins were prescribed in patients with obesity (*p* < 0.10).

The groups did not significantly differ in the frequency of myocardial infarction, stroke, death, and restenosis as late adverse outcomes of CEA (Table 3).

According to the results of the multivariate regression analysis (Table 4), in comparison to patients with a normal BMI, those who were overweight significantly differed in sex, being more frequently males (*p* = 0.004), they more frequently had NIDDM (*p* = 0.023), and the use of ACEI at the time of discharge was more frequent (*p* = 0.003). In comparison to patients with a normal weight, patients with obesity were younger (*p* = 0.029), they more frequently had a myocardial infarction (*p* = 0.002), NIDDM (*p* < 0.001), and increased triglyceride levels (*p* = 0.045). Moreover, patients with obesity more frequently used OAC in the preoperative therapy (*p* = 0.010), and the clump duration was shorter (*p* = 0.023).

In the ROC curve analysis, there was no significant correlation between the level of the BMI and any of the late adverse outcomes [myocardial infarction (area under the curve—AUC = 0.502, *p* = 0.955), stroke (AUC = 0.502, *p* = 0.966), death (AUC = 0.484, *p* = 0.583), and restenosis (AUC = 0.516, *p* = 0.563)]. Moreover, there was no significant correlation between the independent variables included in the study. None of the correlation coefficients was higher than 0.75, taken as the limit value significant for multicollinearity. The follow-up data concerning stroke, myocardial infarction, and death, separately and together, did not show any difference between those with a normal weight, those who were overweight, and those with obesity, analyzed by the Kaplan–Meier test. The curves almost overlapped with *p* values ranging from 0.973 to 0.764. (The data are available as supplementary materials).

According to a Cox regression analysis (Table 5), patients with and patients without late major adverse outcomes (myocardial infarction, stroke, and death), analyzed separately or altogether, did not differ in the BMI. In the logistic regression analysis, being overweight and having obesity were also not predictors for restenosis (Table 5).

## 4. Discussion

In the present investigation, there were no significant differences in the frequency of late adverse outcomes after CEAs in patients who were overweight and patients with obesity compared separately to the CEAs in patients with a normal BMI.

As already stated, obesity is considered a risk factor for many diseases, including cardiovascular and cerebrovascular diseases [1,2,3,4,5,6], as well as a risk of perioperative mortality in patients undergoing vascular surgery, including CEA [7,8].

There is a large amount of literature data about the association between the BMI and early adverse outcomes after CEA [13,15,16,18], but the results have been inconsistent.

The literature data on the association of BMI with late adverse outcomes after CEA are scarce. In fact, we found only a few articles [16,20,21,22]. When early adverse outcomes after CEA were considered, our results were similar to the results of these studies. In the analysis of the early adverse outcomes after CEA [14], we found that being overweight and having obesity were associated neither with major nor minor complications nor the need for reoperation. Only bleeding was significantly less frequent after CEA in patients who were overweight compared to patients with a normal weight. In Volkers et al.’s study [16], BMI was not associated with postprocedural risk of stroke or death, and in the study by Jeong et al. [21], BMI was not associated with early major adverse events (MAEs). In the study of Arinze et al. [20] conducted on nearly 90,000 patients, only those with morbid obesity (BMI ≥ 40 kg/m^2^) had significantly higher perioperative cardiac complications, while bleeding was significantly more frequent in patients who were underweight; however, neither of these two BMI groups was analyzed separately in our study. However, while we did not find an association between being overweight and having obesity with any of the late adverse outcomes, Volkers et al. [16] found that a BMI 25–29.9 was associated with a lower postprocedural risk of stroke or death than a BMI 20-24.9. In the study by Arinze et al. [20], one-year and five-year survival after carotid endarterectomy was significantly associated with the body mass index. Compared to those with a normal weight, patients who were underweight had an increased risk for one-year mortality, but in both patients who were overweight and patients with obesity, the risk for one-year mortality was decreased. The same associations persisted at the five-year time point. Moreover, compared to patients with a normal weight, those with morbid obesity had a decreased risk for five-year mortality. The authors stated that these results agreed with the questionable obesity paradox [23], also present in the study by Volkers et al. [16]. In the recent study by Blecha et al. [22], a BMI < 20 kg/m^2^ was one of the predictors of five-year mortality after CEA for asymptomatic carotid stenosis. In the present investigation, we analyzed symptomatic and asymptomatic patients together, and we did not include patients who were underweight in the analysis due to the very small number. Jeong et al. [21] did not find an association between the BMI and late MAEs but found that a higher BMI was significantly related to the occurrence of restenosis. It is difficult to say whether these differences in the association between the BMI and late complications after CEA are the results of the fact that our sample was small in comparison with some other studies or whether they occurred as a result of some differences in the variables in terms of the potential confounders that were included in these investigations.

Although being overweight and having obesity were not significantly related to late complications after CEA, they were, significantly and independently of other variables, associated with some factors found to be predictors of late adverse outcomes, such as non-insulin-dependent diabetes mellitus [24,25,26,27,28], increased triglyceride levels [29], and some other cardiovascular diseases in the personal history, according to the patients’ reports or postulated based on the therapy they received [30,31,32].

In the present study, non-insulin-dependent diabetes mellitus was significantly more frequent in both patients who were overweight and patients with obesity, compared to those with a normal weight. It is well known that an increased BMI is strongly correlated with type 2 diabetes mellitus (T2DM) [33,34]. According to a meta-analysis from the USA and Europe, people with obesity had a several times higher chance of developing T2DM compared to those with a normal weight [35]. The fat tissue distribution is considered the crucial factor in developing insulin resistance and, consequently, T2DM, independent from the stage of obesity [36], and those with a high proportion of visceral fat and limited abdominal subcutaneous fat are more insulin-resistant [37].

Both obesity and atherosclerosis are lipid storage disorders, with triglyceride accumulation in the fat tissue and cholesterol esters in atherosclerotic plaques [38]. In our study, the patients with obesity had significantly higher triglyceride levels than the patients with a normal weight. High triglyceride (TG) levels reflect the presence of high levels of TG-rich lipoprotein (TRL) remnants [39], which seem to be more proatherogenic than LDLs [40]. The accumulation of TRL remnants in atherosclerotic plaques plays an important role in the inflammatory response and the further development of atherosclerosis [41]. Hypertriglyceridemia, as secondary dyslipidemia in obesity, may contribute to the formation and progression of atherosclerotic plaques, including the carotid district. A recent review article demonstrates that both fasting and non-fasting hypertriglyceridemia are risk factors for CAS progression and cerebrovascular events associated with CAS [42].

It is also known that obesity contributes directly to incident cardiovascular risk factors, including dyslipidemia, type 2 diabetes, hypertension, and sleep disorders, but it can lead to the development of some cardiovascular diseases and cardiovascular disease mortality, independent from other risk factors [43,44]. The susceptibility to obesity-related cardiovascular diseases is not mediated solely by the total body fat mass but also depends on the individual differences in regional body fat distribution. The cardiovascular complications associated with obesity are also driven by various mechanisms, such as adipocytokines imbalance, inflammation, insulin resistance, endothelial dysfunction, coronary calcification, activation of coagulation, renin angiotensin, or the sympathetic nervous systems [45]. Powell-Wiley et al. stressed the need for further evaluation of the mechanisms underlying obesity-related cardiac dysfunction.

There were some limitations to our investigation. Due to the small numbers, patients who were underweight were not included in the present study. Moreover, class II and III obesity were put together with class I obesity, and all these BMI subgroups could be, according to the results of other investigations, associated with complications after CEA. There is also the question of whether BMI is the best measure of adiposity, or whether it would be better to use measures such as the waist circumference, waist-to-height ratio, waist-to-thigh ratio, or the InBody Test [46,47,48,49].

## 5. Conclusions

Compared to the patients with a normal weight, the patients who were overweight were significantly more frequently males, with non-insulin-dependent diabetes mellitus, and a more frequent use of ACEI in hospital discharge therapy. The patients with obesity were significantly younger, with myocardial infarction and non-insulin-dependent diabetes mellitus in their personal history, with more frequently increased triglyceride levels, more frequent usage of OAC in the therapy before the operation, and a shorter clump duration.

However, being overweight or having obesity was not significantly associated with the occurrence of myocardial infarction, stroke, death, and restenosis, as the late adverse outcomes after a carotid endarterectomy.

## Figures and Tables

**Table 1:** Demographic characteristics, smoking, personal history, therapy, laboratory values on admission, and family history of cardiovascular diseases, by body mass index, according to the univariate logistic regression analysis.

Body Mass Index Variable	Normal Weight 18.5–24.9 kg/m^2^ *n* = 413	Overweight 25.0–29.9 kg/m^2^ *n* = 583	Obese ≥30 kg/m^2^ *n* = 220
Age, y, mean ± SD	68.41 ± 8.17	68.10 ± 7.29	66.87 ± 7.87 ^c^
Sex—Male, *n* (%)	229 (55.4)	379 (65.0) ^b^	117 (53.2)
Smoking, *n* (%)	205 (49.6)	267 (45.8)	98 (44.5)
Personal history, *n* (%):			
Myocardial infarction	21 (5.1)	40 (6.9)	26 (11.8) ^b^
PCI	26 (6.3)	39 (6.7)	12 (5.5)
ACB	26 (6.3)	54 (9.3) ^d^	20 (9.1)
Chronic heart failure	8 (1.9)	10 (1.7)	3 (1.4)
Peripheral arterial disease	56 (13.6)	97 (16.6)	37 (16.8)
Aneurysmatic disease	9 (2.2)	25 (4.3) ^d^	6 (2.7)
Hyperlipoproteinemia	372 (90.1)	524 (89.9)	196 (89.1)
Hypertension	386 (93.5)	543 (93.1)	209 (95.0)
IDDM	29 (7.0)	43 (7.4)	21 (9.5)
NIDDM	73 (17.7)	137 (23.5) ^c^	68 (30.9) ^a^
Therapy before the operation, *n* (%):			
Aspirin	334 (80.9)	483 (82.8)	182 (82.7)
Clopidogrel	117 (28.3)	157 (26.9)	60 (27.3)
OAC	14 (3.4)	26 (4.5)	17 (7.7) ^c^
ACEI	296 (71.7)	425 (72.9)	166 (75.5)
β blockers	184 (44.6)	284 (48.7)	108 (49.1)
Statin	269 (65.1)	378 (64.8)	137 (62.3)
Laboratory values on admission, *n* (%):			
Cholesterol ≥ 5.2 mmol/L	206 (49.9)	275 (47.2)	98 (44.5)
Triglycerides ≥ 1.7 mmol/L	202 (48.9)	309 (53.0)	133 (60.5) ^c^
Family history of CVD	182 (44.1)	270 (46.3)	113 (51.4) ^d^

ACB—aortocoronary bypass; ACEI—angiotensin-converting enzyme inhibitors; CVD—cardiovascular disease; OAC—oral anticoagulant; PCI—percutaneous coronary intervention; IDDM—insulin-dependent diabetes mellitus; NIDDM—non-insulin-dependent diabetes mellitus. ^a^ *p* < 0.001; ^b^ *p* ≤ 0.01; ^c^ *p* < 0.05; ^d^ *p* ≤ 0.10.

**Table 2:** Characteristics of carotid disease, operative data, and the hospital discharge therapy by body mass index, according to the univariate logistic regression analysis.

Body Mass Index Variable	Normal Weight 18.5–24.9 kg/m^2^ *n* = 413	Overweight 25.0–29.9 kg/m^2^ *n* = 583	Obese ≥30 kg/m^2^ *n* = 220
Characteristics of carotid disease, *n* (%):			
Symptomatic	135 (32.7)	212 (36.4)	79 (35.9)
Complicated plaque	63 (15.2)	95 (16.3)	29 (13.2)
Ipsilateral stenosis, *n* (%):			
50–69%	60 (14.5)	85 (14.6)	40 (18.2)
70–89%	235 (56.9)	329 (56.4)	103 (45.8)
90–99%	117 (28.3)	168 (28.8)	74 (33.6)
Contralateral stenosis, *n* (%):			
50–69%	76 (18.4)	97 (16.6)	33 (15.0)
70–89%	41 (9.9)	57 (9.8)	17 (7.7)
90–99%	23 (5.6)	22 (3.8)	5 (2.3)
100%	28 (6.8)	48 (8.2)	26 (11.8)
Operative data, *n* (%):			
Urgent endarterectomy	6 (1.5)	6 (1.0)	2 (1.0)
Clamp duration			
<10 min	71 (17.4)	114 (19.8)	50 (22.7)
10–15 min	258 (63.4)	367 (63.7)	135 (61.4)
>15 min	78 (19.2)	95 (16.5)	35 (15.9) ^c^
Hospital discharge therapy, *n* (%):			
Aspirin	404 (97.8)	563 (96.6)	216 (98.2)
Clopidogrel	288 (69.7)	407 (69.8)	150 (68.2)
OAK	12 (2.9)	24 (4.1)	14 (6.4) ^b^
ACEI	306 (74.1)	477 (81.8) ^a^	172 (78.2)
β blockers	219 (53.0)	324 (55.6)	129 (58.6)
Statin (dose):			
0	13 (3.1)	20 (3.4)	3 (1.4)
10 mg	19 (4.6)	26 (4.5)	6 (2.7)
20 mg	376 (91.0)	534 (91.6)	208 (94.5)
40 mg	5 (1.2)	3 (0.5)	3 (1.4) ^c^

ACEI—angiotensin-converting enzyme inhibitors; OAC—oral anticoagulant. ^a^ *p* < 0.01; ^b^ *p* < 0.05; ^c^ *p* ≤ 0.10.

**Table 3:** Late adverse outcomes after carotid endarterectomies *.

Body Mass Index	Normal Weight 18.5–24.9 kg/m^2^ *n* = 413	Overweight 25.0–29.9 kg/m^2^ *n* = 583	Obese ≥30 kg/m^2^ *n* = 220
Complications—*n* (%):			
Myocardial infarction	16 (3.9)	27 (4.6)	11 (5.0)
Stroke	23 (5.6)	32 (5.5)	13 (5.9)
Death	18 (4.4)	30 (5.1)	12 (5.5)
Restenosis **	42 (10.6)	55 (9.9)	24 (11.5)

* According to univariate logistic regression analysis. ** Data for restenosis were available for 395 CEAs in patients with a normal BMI, 553 CEAs in patients who were overweight, and 208 CEAs in patients with obesity.

**Table 4:** Carotid endarterectomies—significant differences between the compared body mass index groups, according to the multivariate logistic regression analysis.

Variable	Odds Ratio	95% Confidence Interval	*p* Value
Patients who were overweight vs. patients with a normal BMI			
Sex—males	1.46	1.12–1.89	0.004
NIDDM	1.45	1.05–2.00	0.023
ACEI as the hospital discharge therapy	1.59	1.17–2.16	0.003
Patients with obesity vs. patients with a normal BMI			
Age	0.98	0.96–1.00	0.029
Myocardial infarction in the personal history	2.69	1.42–5.08	0.002
NIDDM	2.13	1.42–3.18	<0.001
OAC therapy before the operation	2.80	1.27–6.11	0.010
Triglyceride level on admission ≥ 1.7 mmol/L	1.43	1.01–2.03	0.045
Clump duration	0.72	0.54–0.96	0.023

ACEI—angiotensin-converting enzyme inhibitors; BMI—body mass index; OAC—oral anticoagulants; NIDDM—non-insulin-dependent diabetes mellitus.

**Table 5:** Association of being overweight and having obesity with the occurrence of late major adverse outcomes (myocardial infarction, stroke, and death) and restenosis after carotid endarterectomies *.

Body Mass Index Subgroups ^a^	Late Major Adverse Outcomes after CEAs		
	NO (*n* = 1094)	YES (*n* = 122)	*p*
	*n* (90.0%)	*n* (10.0%)	Values *
Normal—referent category	374 (34.2)	39 (32.0)	
Overweight	524 (47.9)	59 (48.4)	0.710
Obese	196 (17.9)	24 (19.7)	0.548
Body mass index subgroups ^a^	Myocardial infarction after CEAs		
	NO (*n* = 1162)	YES (*n* = 54)	*p*
	*n* (95.6%)	*n* (4.4%)	values *
Normal—referent category	397 (34.2)	16 (29.6)	
Overweight	556 (47.8)	27 (50.0)	0.556
Obese	209 (18.0)	11 (20.4)	0.505
Body mass index subgroups ^a^	Stroke after CEAs		
	NO (*n* = 1148)	YES (*n* = 68)	*p*
	*n* (94.4%)	*n* (5.6%)	values *
Normal—referent category	390 (34.0)	23 (33.8)	
Overweight	551 (48.0)	32 (47.1)	0.856
Obese	207 (18.0)	13 (19.1)	0.959
Body mass index subgroups ^a^	Death after CEAs		
	NO (*n* = 1195)	YES (*n* = 101)	*p*
	*n* (92.2%)	*n* (7.8%)	values *
Normal—referent category	409 (34.2)	36 (35.6)	
Overweight	572 (47.9)	46 (45.5)	0.563
Obese	214 (17.9)	19 (18.8)	0.529
Body mass index subgroups ^a^	Restenosis after CEAs		
	NO (*n* = 1035)	YES (*n* = 121)	*p*
	*n* (89.5%)	*n* (10.5%)	values **
Normal—referent category	353 (34.1)	42 (34.7)	
Overweight	498 (48.1)	55 (45.5)	0.810
Obese	184 (17.8)	24 (19.8)	0.830

^a^ Underweight was not included in the analyses (seven CEAs for MAOs, seven for myocardial infarction, and seven for stroke, nine for death, and six CEAs for restenosis). * According to the univariate Cox regression analysis. ** According to the univariate logistic regression analysis.

## Data Availability

The database may be provided upon request.

## Supplementary Materials

The following supporting information can be downloaded at: https://www.mdpi.com/article/10.3390/ijerph20032692/s1.
