# Supplementary material for: Body Mass Index and Late Adverse Outcomes after a Carotid Endarterectomy
Source: Int J Environ Res Public Health. 2023 Feb 2;20(3):2692. doi: 10.3390/ijerph20032692 (PMC9916381; doi:10.3390/ijerph20032692)
Supplement: Supplementary file 1 [file ijerph-20-02692-s001.zip › Supplementary File 1.pdf]

A

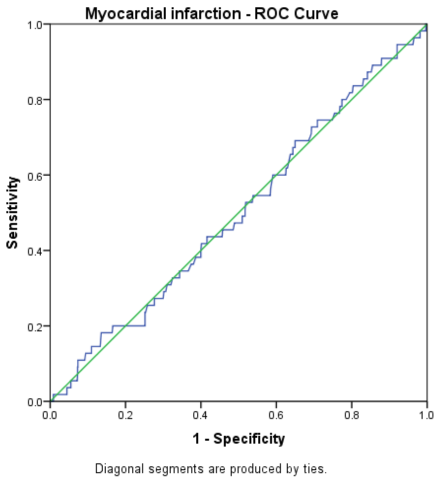

B

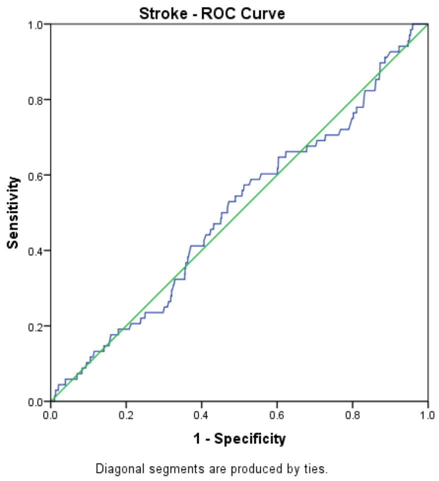

C

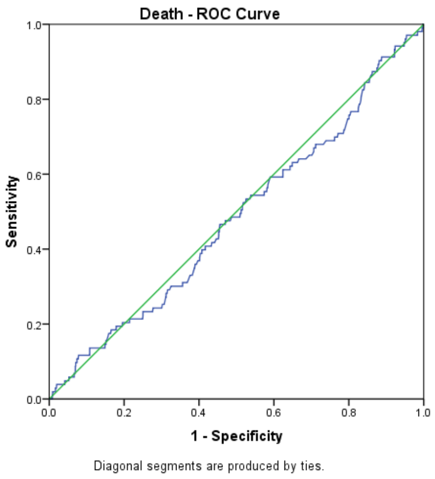

D

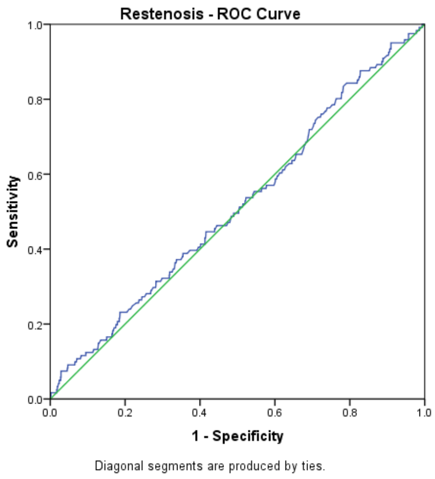

Figure S1. ROC curve analysis of correlation between the level of BMI and the late adverse outcomes after CEA: (A) Myocardial infarction (AUC: 0.502; p=0.955), (B) Stroke (AUC: 0.502; p=0.966), (C) Death (AUC: 0.484; p=0.583), and (D) Restenosis (AUC: 0.516; p=0.563).
