# Supplementary material for: Body Mass Index and Late Adverse Outcomes after a Carotid Endarterectomy
Source: Int J Environ Res Public Health. 2023 Feb 2;20(3):2692. doi: 10.3390/ijerph20032692 (PMC9916381; doi:10.3390/ijerph20032692)
Supplement: Supplementary file 1 [file ijerph-20-02692-s001.zip › Supplementary File 2.pdf]

A

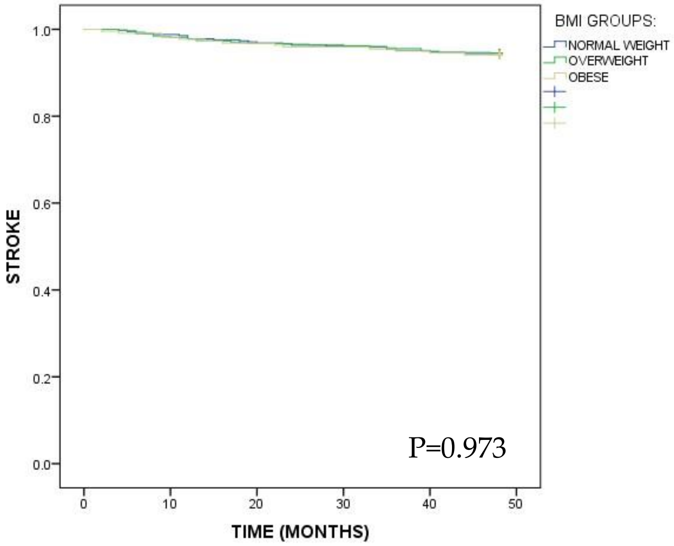

N. at risk	12	24	36	48
NW	404	398	393	390
OW	570	563	557	551
OB	215	211	209	207

B

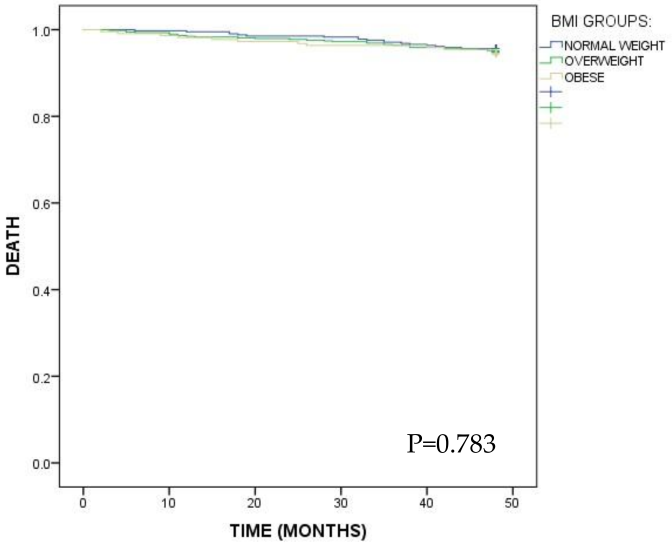

N. at risk	12	24	36	48
NW	411	407	401	395
OW	574	570	560	553
OB	216	214	212	208

C

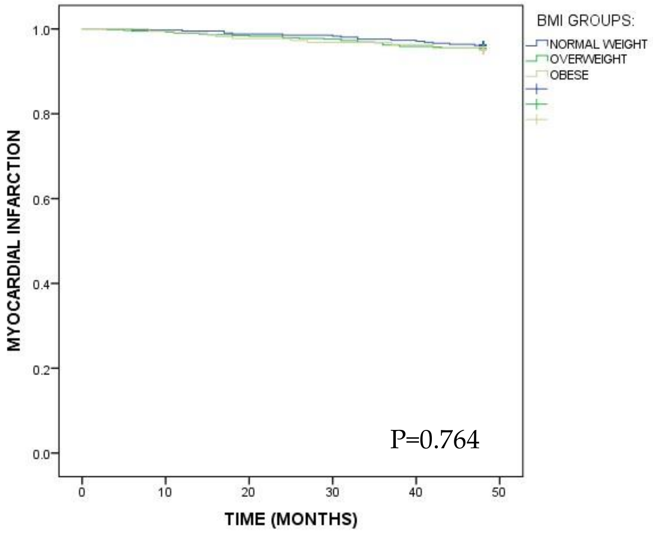

N. at risk	12	24	36	48
NW	411	407	403	397
OW	577	571	561	556
OB	218	215	213	209

D

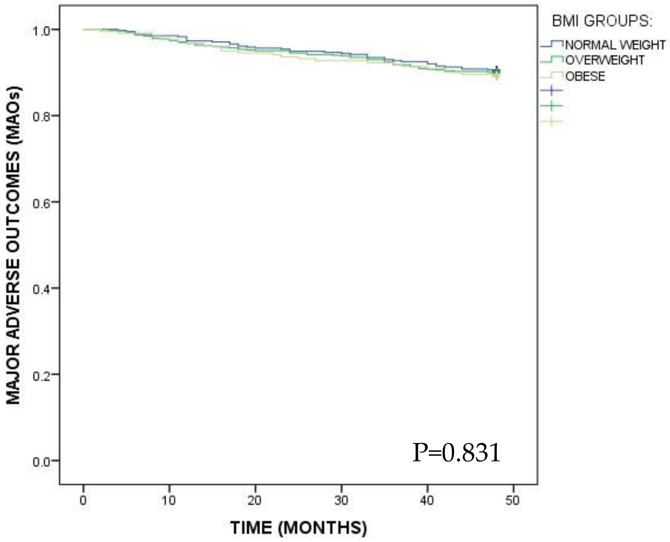

N. at risk	12	24	36	48
NW	402	392	383	374
OW	564	551	535	524
OB	213	206	202	196

Figure S2: Kaplan-Meier analysis for: (A) stroke (p=0.973), (B) death (p=0.783), (C) myocardial infarction (p=0.764), and (D) MAOs (p=0.831).
