# Supplementary material for: Body Mass Index and Late Adverse Outcomes after a Carotid Endarterectomy
Source: Int J Environ Res Public Health. 2023 Feb 2;20(3):2692. doi: 10.3390/ijerph20032692 (PMC9916381; doi:10.3390/ijerph20032692)
Supplement: Supplementary file 1 [file ijerph-20-02692-s001.zip › Supplementary File 3 .pdf]

```
CORRELATIONS
  /VARIABLES=Godine_starosti Pol bmi Prethodniinfarktmiokarda
PrethodnaPCI PethrodniACB
    Zastojnasrcanaslabost Perifernaarterijskabolest Aneurizmatskabolest
Hiperlipoproteinemija
    Hipertenzija PorodicnoopterecenjeKVbolest Dijabetes
  /PRINT=TWOTAIL NOSIG
  /MISSING=PAIRWISE.
```

Correlations

Notes

Output Created		06-JAN-2023 16:49:54
Comments		
Input	Data	/Users/jelenamarinkovic/Desktop/Danka juli 2022/Baza sa 1597 redova - rane. kasne i ostale varijable - poslednje. - 26.07.2022.sav
	Active Dataset	DataSet1
	Filter	<none>
	Weight	<none>
	Split File	<none>
	N of Rows in Working Data File	1597
Missing Value Handling	Definition of Missing	User-defined missing values are treated as missing.
	Cases Used	Statistics for each pair of variables are based on all the cases with valid data for that pair.

Syntax		CORRELATIONS<br><br>/VARIABLES=Godine_starosti Pol bmi Prethodniinfarktmiokarda PrethodnaPCI PethrodniACB Zastojnasrcanaslabost Perifernaarterijskabolest Aneurizmatskabolest Hiperlipoproteinemija Hipertenzija PorodicnoopterecenjeKVbolest Dijabetes<br>/PRINT=TWOTAIL NOSIG<br>/MISSING=PAIRWISE.
Resources	Processor Time	00:00:00.02
	Elapsed Time	00:00:00.00

Correlations

		Godine_starosti	Pol	bmi	Prethodniinfarktmiokarda	PrethodnaPCI	PethrodniACB							
Godine_starosti	Pearson Correlation	1	-.069**	-.079**	-.014	.001	.016							
	Sig. (2-tailed)		.006	.002	.583	.979	.528							
	N	1597	1597	1597	1597	1597	1597							
Pol	Pearson Correlation	-.069**	1	-.032	-.075**	-.044	-.063*							
	Sig. (2-tailed)	.006		.198	.003	.081	.012							
	N	1597	1597	1597	1597	1597	1597							
bmi	Pearson Correlation	-.079**	-.032	1	.033	-.004	.038							

	Sig. (2-tailed)	.002	.198		.183	.859	.133							
	N	1597	1597	1597	1597	1597	1597							
Prethodniinfarktmiokarda	Pearson Correlation	-.014	-.075**	.033	1	.190**	.147**							
	Sig. (2-tailed)	.583	.003	.183		.000	.000							
	N	1597	1597	1597	1597	1597	1597							
PrethodnaPCI	Pearson Correlation	.001	-.044	-.004	.190**	1	.043							
	Sig. (2-tailed)	.979	.081	.859	.000		.087							
	N	1597	1597	1597	1597	1597	1597							
PethrodniACB	Pearson Correlation	.016	-.063*	.038	.147**	.043	1							
	Sig. (2-tailed)	.528	.012	.133	.000	.087								
	N	1597	1597	1597	1597	1597	1597							
Zastojnasrcanaslabost	Pearson Correlation	.042	-.064*	-.002	.212**	.046	.180**							
	Sig. (2-tailed)	.094	.010	.948	.000	.065	.000							
	N	1597	1597	1597	1597	1597	1597							
Perifernaarterijskabolest	Pearson Correlation	-.060*	-.056*	.000	.071**	.046	-.027							
	Sig. (2-tailed)	.016	.025	.995	.004	.064	.281							
	N	1597	1597	1597	1597	1597	1597							

Aneurizmatska bolest	Pearson Correlation	-.008	-.064*	.007	.024	.061*	.045							
	Sig. (2-tailed)	.763	.011	.777	.339	.015	.071							
	N	1597	1597	1597	1597	1597	1597							
Hiperlipoproteinemija	Pearson Correlation	.022	.080**	-.004	-.012	.059*	.051*							
	Sig. (2-tailed)	.371	.001	.884	.640	.018	.041							
	N	1597	1597	1597	1597	1597	1597							
Hipertenzija	Pearson Correlation	.055*	.090**	.028	-.004	.029	.032							
	Sig. (2-tailed)	.027	.000	.263	.862	.255	.206							
	N	1597	1597	1597	1597	1597	1597							
PorodicnoopterecenjeKVbolest	Pearson Correlation	-.041	.108**	.038	.072**	.033	.055*							
	Sig. (2-tailed)	.103	.000	.134	.004	.193	.028							
	N	1597	1597	1597	1597	1597	1597							
Dijabetes	Pearson Correlation	-.030	-.008	.139**	.058*	.032	.070**							
	Sig. (2-tailed)	.236	.763	.000	.020	.201	.005							
	N	1597	1597	1597	1597	1597	1597							

Correlations

	Zastojnasrcanaslabost	Periferna arterijska bolest	Aneurizmatskabolest	Hiperlipoproteinemija	Hipertenzija	PorodicnoopterecenjeKVbolest	

Godine_starosti	Pearson Correlation	.042	-.060*	-.008	.022	.055*	-.041	
	Sig. (2-tailed)	.094	.016	.763	.371	.027	.103	
	N	1597	1597	1597	1597	1597	1597	
Pol	Pearson Correlation	-.064*	-.056*	-.064*	.080**	.090**	.108**	
	Sig. (2-tailed)	.010	.025	.011	.001	.000	.000	
	N	1597	1597	1597	1597	1597	1597	
bmi	Pearson Correlation	-.002	.000	.007	-.004	.028	.038	
	Sig. (2-tailed)	.948	.995	.777	.884	.263	.134	
	N	1597	1597	1597	1597	1597	1597	
Prethodniinfarktmiokarda	Pearson Correlation	.212**	.071**	.024	-.012	-.004	.072**	
	Sig. (2-tailed)	.000	.004	.339	.640	.862	.004	
	N	1597	1597	1597	1597	1597	1597	
PrethodnaPCI	Pearson Correlation	.046	.046	.061*	.059*	.029	.033	
	Sig. (2-tailed)	.065	.064	.015	.018	.255	.193	
	N	1597	1597	1597	1597	1597	1597	
PethrodniACB	Pearson Correlation	.180**	-.027	.045	.051*	.032	.055*	
	Sig. (2-tailed)	.000	.281	.071	.041	.206	.028	
	N	1597	1597	1597	1597	1597	1597	
Zastojnasrcanaslabost	Pearson Correlation	1	.040	.067**	.029	.014	.090**	
	Sig. (2-tailed)		.114	.008	.251	.579	.000	
	N	1597	1597	1597	1597	1597	1597	
Perifernaarterijskabolest	Pearson Correlation	.040	1	.193**	.011	.009	.094**	
	Sig. (2-tailed)	.114		.000	.652	.708	.000	
	N	1597	1597	1597	1597	1597	1597	
Aneurizmatskabolest	Pearson Correlation	.067**	.193**	1	-.001	.045	.030	
	Sig. (2-tailed)	.008	.000		.982	.069	.236	
	N	1597	1597	1597	1597	1597	1597	

Hiperlipoproteinemija	Pearson Correlation	.029	.011	-.001	1	.277**	.084**	
	Sig. (2-tailed)	.251	.652	.982		.000	.001	
	N	1597	1597	1597	1597	1597	1597	
Hipertenzija	Pearson Correlation	.014	.009	.045	.277**	1	.125**	
	Sig. (2-tailed)	.579	.708	.069	.000		.000	
	N	1597	1597	1597	1597	1597	1597	
PorodicnoopterecenjeKV bolest	Pearson Correlation	.090**	.094**	.030	.084**	.125**	1	
	Sig. (2-tailed)	.000	.000	.236	.001	.000		
	N	1597	1597	1597	1597	1597	1597	
Dijabetes	Pearson Correlation	.053*	.091**	-.026	.062*	.056*	.021	
	Sig. (2-tailed)	.034	.000	.296	.013	.026	.392	
	N	1597	1597	1597	1597	1597	1597	

Correlations

		Dijabetes
Godine_starosti	Pearson Correlation	-.030
	Sig. (2-tailed)	.236
	N	1597
Pol	Pearson Correlation	-.008
	Sig. (2-tailed)	.763
	N	1597
bmi	Pearson Correlation	.139**
	Sig. (2-tailed)	.000
	N	1597
Prethodniinfarktmiokarda	Pearson Correlation	.058*
	Sig. (2-tailed)	.020
	N	1597
PrethodnaPCI	Pearson Correlation	.032
	Sig. (2-tailed)	.201
	N	1597
PethrodniACB	Pearson Correlation	.070**
	Sig. (2-tailed)	.005
	N	1597
Zastojnasrcanaslabost	Pearson Correlation	.053*
	Sig. (2-tailed)	.034
	N	1597
Perifernaarterijskabolest	Pearson Correlation	.091**
	Sig. (2-tailed)	.000

	N	1597
Aneurizmatskabolest	Pearson Correlation	-.026
	Sig. (2-tailed)	.296
	N	1597
Hiperlipoproteinemija	Pearson Correlation	.062*
	Sig. (2-tailed)	.013
	N	1597
Hipertenzija	Pearson Correlation	.056*
	Sig. (2-tailed)	.026
	N	1597
PorodicnoopterecenjeKVbolest	Pearson Correlation	.021
	Sig. (2-tailed)	.392
	N	1597
Dijabetes	Pearson Correlation	1
	Sig. (2-tailed)	
	N	1597

**. Correlation is significant at the 0.01 level (2-tailed).
*. Correlation is significant at the 0.05 level (2-tailed).

```
CORRELATIONS
  /VARIABLES=PUSENJE trigliceridi_kat holesterol_kat SIMPTOMI_AMAUROSIS
SIMPTOMI_TIA
    SIMPTOMI_MOZDANIUDAR simptomatski_ili_ne_pacijenti
  /PRINT=TWOTAIL NOSIG
  /MISSING=PAIRWISE.
```

Correlations

Notes

Output Created		06-JAN-2023 16:51:03
Comments		
Input	Data	/Users/jelenamarinkovic/Desktop/Danka juli 2022/Baza sa 1597 redova - rane. kasne i ostale varijable - poslednje. - 26.07.2022.sav
	Active Dataset	DataSet1
	Filter	<none>
	Weight	<none>

	Split File	<none>
	N of Rows in Working Data File	1597
Missing Value Handling	Definition of Missing	User-defined missing values are treated as missing.
	Cases Used	Statistics for each pair of variables are based on all the cases with valid data for that pair.
Syntax		CORRELATIONS /VARIABLES=PUSENJE trigliceridi_kat holesterol_kat SIMPTOMI_AMAUROSIS SIMPTOMI_TIA SIMPTOMI_MOZDANIUDAR simptomatski_ili_ne_pacijenti /PRINT=TWOTAIL NOSIG /MISSING=PAIRWISE.
Resources	Processor Time	00:00:00.01
	Elapsed Time	00:00:00.00

Correlations

		PUSENJE	trigliceridi_kat	holesterol_kat	SIMPTOMI_AMAUROSIS	SIMPTOMI_TIA		
PUSENJE	Pearson Correlation	1	-.037	.067**	.061*	.011		
	Sig. (2-tailed)		.137	.008	.015	.668		
	N	1597	1584	1584	1597	1597		
trigliceridi_kat	Pearson Correlation	-.037	1	.243**	.018	.002		
	Sig. (2-tailed)	.137		.000	.471	.925		
	N	1584	1584	1584	1584	1584		
holesterol_kat	Pearson Correlation	.067**	.243**	1	-.009	-.003		
	Sig. (2-tailed)	.008	.000		.729	.898		
	N	1584	1584	1584	1584	1584		

SIMPTOMI_AMAUROSIS	Pearson Correlation	.061*	.018	-.009	1	.037		
	Sig. (2-tailed)	.015	.471	.729		.141		
	N	1597	1584	1584	1597	1597		
SIMPTOMI_TIA	Pearson Correlation	.011	.002	-.003	.037	1		
	Sig. (2-tailed)	.668	.925	.898	.141			
	N	1597	1584	1584	1597	1597		
SIMPTOMI_MOZDANIUDAR	Pearson Correlation	.045	.008	-.014	-.052*	-.128**		
	Sig. (2-tailed)	.074	.742	.573	.037	.000		
	N	1597	1584	1584	1597	1597		
simptomatski_ili_ne_pacijenti	Pearson Correlation	.072**	.012	-.008	.239**	.540**		
	Sig. (2-tailed)	.004	.630	.752	.000	.000		
	N	1597	1584	1584	1597	1597		

Correlations

		SIMPTOMI_MOZDANIUDAR	simptomatski_ili_ne_pacijenti
PUSENJE	Pearson Correlation	.045	.072**
	Sig. (2-tailed)	.074	.004
	N	1597	1597
trigliceridi_kat	Pearson Correlation	.008	.012
	Sig. (2-tailed)	.742	.630
	N	1584	1584
holesterol_kat	Pearson Correlation	-.014	-.008
	Sig. (2-tailed)	.573	.752
	N	1584	1584
SIMPTOMI_AMAUROSIS	Pearson Correlation	-.052*	.239**
	Sig. (2-tailed)	.037	.000
	N	1597	1597
SIMPTOMI_TIA	Pearson Correlation	-.128**	.540**
	Sig. (2-tailed)	.000	.000
	N	1597	1597
SIMPTOMI_MOZDANIUDAR	Pearson Correlation	1	.683**
	Sig. (2-tailed)		.000
	N	1597	1597
simptomatski_ili_ne_pacijenti	Pearson Correlation	.683**	1
	Sig. (2-tailed)	.000	
	N	1597	1597

**. Correlation is significant at the 0.01 level (2-tailed).
*. Correlation is significant at the 0.05 level (2-tailed).

```
CORRELATIONS
  /VARIABLES=TERAP_ASPIRIN_preOP TERAP_KLOPIDOGREL_preOP
TERAP_OAK_preOP TERAPIJA_ACEI_preOP
    TERAPIJA_BETABLOK_preOP TERAPIJA_STATINI_preOP IPSILAT_STENOZA
KONTRALAT_STENOZA
  /PRINT=TWOTAIL NOSIG
  /MISSING=PAIRWISE.
```

Correlations

Notes

Output Created		06-JAN-2023 16:51:42
Comments		
Input	Data	/Users/jelenamarinkovic/Desktop/Danka juli 2022/Baza sa 1597 redova - rane. kasne i ostale varijable - poslednje. - 26.07.2022.sav
	Active Dataset	DataSet1
	Filter	<none>
	Weight	<none>
	Split File	<none>
	N of Rows in Working Data File	1597
Missing Value Handling	Definition of Missing	User-defined missing values are treated as missing.
	Cases Used	Statistics for each pair of variables are based on all the cases with valid data for that pair.

Syntax		CORRELATIONS<br><br>/VARIABLES=TERAP_ASPIRIN_preOP TERAP_KLOPIDOGREL_preOP TERAP_OAK_preOP TERAPIJA_ACEI_preOP<br><br>TERAPIJA_BETABLOK_preOP TERAPIJA_STATINI_preOP IPSILAT_STENOZA KONTRALAT_STENOZA<br>/PRINT=TWOTAIL NOSIG<br>/MISSING=PAIRWISE.
Resources	Processor Time	00:00:00.01
	Elapsed Time	00:00:00.00

Correlations

		TERAP_ASPIRIN_preOP	TERAP_KLOPIDOGREL_preOP	TERAP_OAK_preOP	TERAPIJA_ACEI_preOP	TERAPIJA_BETABLOK_preOP			
TERAP_ASPIRIN_preOP	Pearson Correlation	1	.169**	-.103**	.125**	.006			
	Sig. (2-tailed)		.000	.000	.000	.816			
	N	1597	1597	1597	1597	1597			
TERAP_KLOPIDOGREL_preOP	Pearson Correlation	.169**	1	-.048	.018	.042			
	Sig. (2-tailed)	.000		.057	.470	.096			
	N	1597	1597	1597	1597	1597			
TERAP_OAK_preOP	Pearson Correlation	-.103**	-.048	1	.035	.073**			
	Sig. (2-tailed)	.000	.057		.164	.004			
	N	1597	1597	1597	1597	1597			
TERAPIJA_ACEI_preOP	Pearson Correlation	.125**	.018	.035	1	.115**			
	Sig. (2-tailed)	.000	.470	.164		.000			
	N	1597	1597	1597	1597	1597			

TERAPIJA_BETABLOK_preOP	Pearson Correlation	.006	.042	.073**	.115**	1			
	Sig. (2-tailed)	.816	.096	.004	.000				
	N	1597	1597	1597	1597	1597			
TERAPIJA_STATINI_preOP	Pearson Correlation	.249**	.245**	.025	.113**	.163**			
	Sig. (2-tailed)	.000	.000	.316	.000	.000			
	N	1597	1597	1597	1597	1597			
IPSILAT_STENOZA	Pearson Correlation	.036	.025	-.011	-.007	.006			
	Sig. (2-tailed)	.149	.321	.673	.774	.813			
	N	1590	1590	1590	1590	1590			
KONTRALAT_STENOZA	Pearson Correlation	.028	.054*	-.006	-.011	.005			
	Sig. (2-tailed)	.273	.030	.820	.648	.852			
	N	1590	1590	1590	1590	1590			

Correlations

		TERAPIJA_STATINI_preOP	IPSILAT_STENOZA	KONTRALAT_STENOZA
TERAP_ASPIRIN_preOP	Pearson Correlation	.249**	.036	.028
	Sig. (2-tailed)	.000	.149	.273
	N	1597	1590	1590
TERAP_KLOPIDOGREL_preOP	Pearson Correlation	.245**	.025	.054*
	Sig. (2-tailed)	.000	.321	.030
	N	1597	1590	1590
TERAP_OAK_preOP	Pearson Correlation	.025	-.011	-.006
	Sig. (2-tailed)	.316	.673	.820
	N	1597	1590	1590
TERAPIJA_ACEI_preOP	Pearson Correlation	.113**	-.007	-.011
	Sig. (2-tailed)	.000	.774	.648
	N	1597	1590	1590
TERAPIJA_BETABLOK_preOP	Pearson Correlation	.163**	.006	.005
	Sig. (2-tailed)	.000	.813	.852
	N	1597	1590	1590
TERAPIJA_STATINI_preOP	Pearson Correlation	1	.014	-.031
	Sig. (2-tailed)		.582	.216
	N	1597	1590	1590
IPSILAT_STENOZA	Pearson Correlation	.014	1	.087**
	Sig. (2-tailed)	.582		.000
	N	1590	1590	1590
KONTRALAT_STEN	Pearson Correlation	-.031	.087**	1

OZA	Sig. (2-tailed)	.216	.000	
	N	1590	1590	1590

**. Correlation is significant at the 0.01 level (2-tailed).
*. Correlation is significant at the 0.05 level (2-tailed).
